# Clinical roles of autophagy-related proteins Beclin-1 and mTOR in smoking and non-smoking patients with oral leukoplakia

**DOI:** 10.4314/ahs.v23i2.71

**Published:** 2023-06

**Authors:** Ping Zhang, Peng Zhou, Min Yao

**Affiliations:** 1 Department of Stomatology, Children's Hospital of Fudan University, National Children's Medical Center, Shanghai 201102, China; 2 Department of Stomatology, Tongling People's Hospital, Tongling 244002, Anhui Province, China; 3 Department of Stomatology, Affiliated Children's Hospital of Nanjing Medical University, Nanjing 210003, Jiangsu Province, China

**Keywords:** Smoking, oral leukoplakia, autophagy, Beclin-1, mammalian target of rapamycin

## Abstract

**Background:**

To study the expressions of autophagy-related proteins Beclin-1 and mammalian target of rapamycin (mTOR) in smoking and non-smoking patients with oral leukoplakia (OLK).

**Methods:**

A total of 240 patients diagnosed as OLK from January 2017 to December 2017 were enrolled. Beclin-1 and mTOR expressions were detected by immunohistochemistry. Their clinical data were collected. The correlations of smoking with Beclin-1 and mTOR expressions as well as clinical factors were explored by Spearman's analysis.

**Results:**

There were significant differences in gender ratio, age, lesion location, severity and malignancy between smoking and non-smoking OLK patients (P<0.05). The positive expression rate of Beclin-1 in OLK patients with simple hyperplasia and abnormal hyperplasia in the smoking group was significantly lower than that of the non-smoking group (P<0.05). In the abnormal hyperplasia group, the number of cigarettes daily was significantly positively correlated with mTOR expression (*r*=0.843, P=0.042). After the simple hyperplasia group was included, there was a positive correlation between smoking age and positive expression rate of mTOR (*r*=0.942, P=0.012). For number of cigarettes and smoking age, the positive expression rates of Beclin-1 and mTOR showed significant negative correlations (*r*=-0.952, P=0.003, *r*=-0.953, P=0.002).

**Conclusion:**

Autophagy-related proteins Beclin-1 and mTOR may be involved in the smoking-induced pathogenesis of OLK.

## Introduction

Oral squamous cell carcinoma (OSCC) is a common malignant tumour with an increasing incidence rate in the oral and maxillofacial region, and the 5-year survival rate is lower than 50%, so the early diagnosis and treatment for precancerous lesions have attracted widespread attention to prevent OSCC^1^. Oral leukoplakia (OLK) is the most common precancerous lesion, and its incidence rate is up to 2.60%^2^. OLK is caused by multiple factors^3^. Abnormal epithelial hyperplasia is the most typical pathological change, and the lesion is gradually aggravated as the degree of abnormal hyperplasia increases^4^. Smoking plays a crucial role in the onset and progression of OLK^5^, but its effect on the malignant change of OLK remains largely unknown.

The abnormal changes in autophagy have been closely related with tumour biological characteristics^6^. Beclin-1, as an autophagy-related protein, is the mammalian homologue of the yeast autophagy-related gene 7. It is a positive regulator of autophagy, which mainly modulates the aggregation of other autophagy-related proteins on the autophagosome membrane, thereby controlling the formation of autophagosomes and autophagy activity^8^. Besides, mammalian target of rapamycin (mTOR) is a negative regulator in the autophagy pathway^9^.

Therefore, we herein detected the expressions of autophagy-related proteins Beclin-1 and mTOR in smoking and non-smoking patients with OLK using immunohistochemistry, and analysed the risk factors of OLK, aiming to provide approaches for preventing the occurrence of OSCC.

## Materials and Methods

### Sample collection

The data of patients who visited our hospital from January 2017 to December 2017 were retrospectively analysed. Inclusion criteria: a) Patients who were diagnosed as OLK by pathological examination, and b) those who did not receive radiation, chemical or hormone therapy before surgery or biopsy. Exclusion criteria: a) Patients with other immune diseases. A total of 240 patients were enrolled, including 64 cases of simple hyperplasia of OLK, 56 cases of mild epithelial hyperplasia, 70 cases of moderate epithelial hyperplasia, and 40 cases of severe epithelial hyperplasia. The general data of all patients were summarized, including gender, age, smoking history and smoking conditions (number of cigarettes and smoking age), lesion location and malignant change (follow-up deadline: August 2018). The patients were divided into smoking group (history of smoking in the past) and non-smoking group (never actively smoking before biopsy). The smoking age and number of cigarettes smoked per day were recorded. Hyperplasia included simple hyperplasia and abnormal hyperplasia according to pathological changes. Abnormal hyperpasia was classified into mild, moderate and severe hyperplasia according to the degree of malignant change. Based on the results of smoking and histological lesions, the 240 patients were divided into smoking simple hyperplasia group, smoking abnormal hyperplasia group, non-smoking simple hyperplasia group and non-smoking abnormal hyperplasia group. This study was approved by the medical ethics committee of the hospital, and all patients signed the informed consent.

### Main reagents

Rabbit anti-human Beclin-1 polyclonal antibody and mTOR monoclonal antibody were purchased from Shanghai Changdao Biotechnology Co., Ltd. (China). Horseradish peroxide-labeled goat anti-rabbit IgG antibody was provided by Shanghai Hufeng Chemical Co., Ltd. (China). Streptavidin-peroxidase (SP) kit was bought from Abcam (USA). Besides, diaminobenzidine (DAB) colour development kit was purchased from Beijing Zhongshan Golden bridge Biotechnology Co., Ltd. (China).

### Detection of Beclin-1 and mTOR by immunochemistry

The collected tissue samples were fixed, embedded and sliced, followed by deparaffinization with xylene and gradient concentrations of ethanol solutions. Then they were repaired in citrate buffer to fully expose the antigenic determinants. Next, the slides were soaked in 3% hydrogen peroxide at room temperature for 10 min, blocked and added diluted primary antibody, followed by incubation overnight at 4°C. On the next day, they were rinsed, added appropriate amount of biotin-labeled secondary antibody, incubated at room temperature for 30 min, and washed. Subsequently, appropriate amount of DAB was added for 2-5 min of reaction which was terminated using deionized water. Afterwards, the slides were counterstained with hematoxylin for 1.5-2 min, washed, and dehydrated in gradient concentrations of ethanol solutions, followed by soaking in xylene, drying and sealing with neutral gum. Finally, the slides were observed under a microscope to record the experimental results.

### Determination criteria for Beclin-1 and mTOR protein expressions

Blinded evaluation was performed for the pathological slides by two experienced pathologists. Five high-power visual fields (×400) were randomly selected from each slide and observed under a light microscope. The results were determined in accordance with the staining intensity and proportion of positive cells. The staining intensity was scored as follows: 0 point for no colour development, 1 point for light yellow or yellow, 2 points for brownish yellow, and 3 points for brown. Score was recorded based on the positive proportion of stained cells: 0-10% as 1 point, 11-50% as 2 points, and 51-100% as 3 points. The above two scores were multiplied: 0-3 points were defined as negative, and ≥4 points were positive.

### Statistical analysis

SPSS 16.0 software was used for statistical analysis. The Chi-square (χ^2^) test was utilized for the comparisons of expressions of Beclin-1 and mTOR in OLK tissues as well as clinical factors. The correlations were explored by Spearman's analysis. Two-tailed P<0.05 was considered statistically significant.

## Results

### Smoking status and clinical factors of patients

Among the OLK patients, there were 194 smokers and 46 non-smokers. In the simple hyperplasia group, the median number of cigarettes per day was 23, and the median years of smoking were 27. In the abnormal hyperplasia group, the median number of cigarettes per day was 27, and the median years of smoking were 47.

In terms of age, the patients aged 50 years old and above accounted for a larger proportion of the smoking group (simple hyperplasia or abnormal hyperplasia), while those aged below 50 years old had a larger proportion in the non-smoking group (simple hyperplasia or abnormal hyperplasia), with a significant difference (P<0.05).

In terms of gender, the male-female ratio was significantly different between non-smoking and smoking groups (P<0.05), and the men were mostly in the smoking group. The tongue and mouth floor were more prone to lesions in patients with abnormal hyperplasia in the smoking group and patients with simple hyperplasia in the non-smoking group.

Abnormal hyperplasia was more serious in smokers, dominated by moderate-severe abnormal hyperplasia, while non-smokers mostly had mild abnormal hyperplasia. The malignant transformation rate of the smoking group was significantly higher than that of the non-smoking group (P<0.05) (Table 1).

**Table 1:** Smoking status and clinical factors of patients

Clinical factor	Smoking group (n=194)		χ^2^	P	Non-smoking group (n=46)		χ^2^	P
	Simple hyperplasia (n=42)	Abnormal hyperplasia (n=152)			Simple hyperplasia (n=22)	Abnormal hyperplasia (n=24)		
Years of smoking/years	27 (10-53)	47 (18-58)						
Number of	23 (5-43)	27 (10-45)						
cigarettes (pcs/d)								
Age/Y	47 (25-70)	55 (43-79)			51 (38-83)	65 (32-85)		
<50	11 (26.19)	20 (13.16)	4.163	0.041	12 (54.55)	20 (83.33)	4.493	0.034
≥50	31 (73.81)	132 (86.84)			10 (45.45)	4 (16.67)		
Gender			6.244	0.012			6.002	0.014
Male	33 (78.57)	140 (92.11)			5 (22.73)	14 (58.33)		
Female	9 (11.43)	12 (7.89)			17 (77.23)	10 (41.67)		
Lesion location			56.860	<0.001			3.994	0.046
Tongue and mouth floor	14 (33.33)	135 (88.82)			18 (81.82)	13 (54.17)		
Others	28 (66.67)	17 (11.18)			4 (18.18)	11 (45.83)		
Lesion severity								
Mild		38 (25.00)				18 (75.00)		
Moderate-severe		114 (75.00)				6 (25.00)		
Malignant transformation			5.133	0.023			3.994	0.046
Yes	26 (61.90)	120 (78.95)			4 (18.18)	11 (45.83)		
No	16 (38.10)	32 (21.05)			18 (81.82)	13 (54.17)		

### Associations of Beclin-1 and mTOR expressions with smoking

The positive expression rate of Beclin-1 in OLK patients with simple hyperplasia and abnormal hyperplasia in the smoking group was significantly lower than that of the non-smoking group, while the positive expression rate of mTOR was significantly higher in the smoking group than that in the non-smoking group (P<0.05) (Table 2).

**Table 2:** Associations of Beclin-1 and mTOR expressions with smoking

	Beclin-1		mTOR	
	
	Simple hyperplasia	Abnormal hyperplasia	Simple hyperplasia	Abnormal hyperplasia
Smoking group (n=194)	5 (11.90)	10 (6.57)	39 (92.86)	110 (72.37)
Non-smoking group (n=46)	16 (72.73)	16 (66.67)	7 (31.82)	6 (25.00)
χ^2^	24.227	14.323	26.609	20.698
P	<0.001	<0.001	<0.001	<0.001

In patients with mild and moderate-severe abnormal hyperplasia in the smoking group, the positive expression rate of Beclin-1 was significantly lower than that of the non-smoking group, and it was the highest in patients with mild abnormal hyperplasia. The positive expression rate of mTOR was significantly higher in the smoking group than that in the non-smoking group, and it was the highest in patients with moderate-severe abnormal hyperplasia (P<0.05) (Table 3).

**Table 3:** Beclin-1 and mTOR expressions in patients with abnormal hyperplasia

	Beclin-1		mTOR	
	
	Mild abnormal hyperplasia	Moderate-severe abnormal hyperplasia	Mild abnormal hyperplasia	Moderate-severe abnormal hyperplasia
Smokers with abnormal hyperplasia (n=152)	4 (10.53)	6 (5.26)	20 (52.63)	90 (78.95)
Non-smokers with abnormal hyperplasia (n=24)	14 (77.78)	2 (33.33)	4 (22.22)	2 (33.33)
χ^2^	25.328	7.218	4.612	6.630
P	<0.001	0.007	0.032	0.010

### Correlations between number of cigarettes and expressions of Beclin-1 and mTOR

In the abnormal hyperplasia group, the number of cigarettes per day was not significantly correlated with Beclin-1 expression (r=-0.811, P=0.063), but significantly positively correlated with mTOR expression (r=0.843, P=0.042). No correlation was observed between years of smoking and expressions of Beclin-1 and mTOR in the abnormal hyperplasia group. After the simple hyperplasia group was included, there was a positive correlation between years of smoking and positive expression rate of mTOR (r=0.942, P=0.012).

Based on the number of cigarettes and years of smoking, the positive expression rates of Beclin-1 and mTOR had significant negative correlations with each other (r=-0.952, P=0.003; r=-0.953, P=0.002), showing significant differences (P<0.05) (Table 4, Table 5).

**Table 4:** Correlations between number of cigarettes and expressions of Beclin-1 and mTOR

Number of cigarettes per day (X)	Abnormal hyperplasia group (n=152)		Smoking group (n=194)	

	Beclin-1	mTOR	Beclin-1	mTOR
0<X≤10	(2/28)	(13/28)	(4/37)	(20/37)
10<X≤20	(2/30)	(20/30)	(3/39)	(27/39)
20<X≤30	(2/29)	(23/29)	(3/39)	(30/39)
30<X≤40	(2/31)	(25/31)	(3/39)	(34/39)
40<X≤50	(2/32)	(29/32)	(2/40)	(38/40)
r	-0.811	0.843	-0.809	0.823
*P*	0.063	0.042	0.053	0.039

**Table 5:** Correlations between years of smoking and expressions of Beclin-1 and mTOR

Year of smoking (X)	Abnormal hyperplasia group (n=152)		Smoking group (n=194)	

	Beclin-1	mTOR	Beclin-1	mTOR
0<X≤20	(2/29)	(18/29)	(3/38)	(20/38)
20<X≤30	(3/31)	(20/31)	(3/38)	(26/38)
30<X≤40	(1/28)	(22/28)	(2/40)	(33/40)
40<X≤50	(2/32)	(24/32)	(4/38)	(32/38)
50<X≤60	(2/32)	(26/32)	(3/40)	(38/40)
r	0.716	0.743	0.733	0.942
*P*	0.068	0.054	0.062	0.012

## Discussion

Smoking has been closely related to the onset and progression of OLK^10^, and clinical data such as age, gender and pathogenic site can be used to predict the risk of malignant transformation of OLK^11^. Beclin-1 and mTOR play key roles in the autophagy pathway, and their overexpression or deletion may indicate the potential of malignant cell transformation^12^. Therefore, analysing the difference between the expressions of Beclin-1 and mTOR in OLK is helpful to determine the process of occurrence and malignant transformation of oral carcinoma. Autophagy is divided into four stages: initiation, elongation, maturation and degradation. Beclin-1 plays an important role in the initiation stage. It forms trimers with PI3K and Atg4, and continuously recruits autophagy proteins, thereby activating autophagy. The Beclin-1 level has a significant positive correlation with the degree of autophagy^13^. Therefore, Beclin-1 is a specific marker for autophagy. It is well known that PI3K/AKT/mTOR is a classic pathway during tumorigenesis and development, and its excessive activation is closely related to the malignant behaviours of tumours such as invasion, metastasis and proliferation. In this pathway, mTOR protein, as a central regulator of autophagy, can reversely regulate the formation of autophagosomes^14^. The protein expressions of Beclin-1 and mTOR in precancerous lesions and tumours have great differences^15^,^16^, and a higher mTOR protein expression level in OLK patients indicates a higher degree of malignancy^17^. However, there are few studies on OLK among smokers and non-smokers at present.

In this study, in the smoking group, the number of cigarettes per day and years of smoking (27 pieces of cigarettes, 47 years) in patients with abnormal hyperplasia significantly exceeded those of patients with simple hyperplasia (23 pieces of cigarettes, 27 years). There were more OLK patients aged 50 years old and above in the smoking group, while there were more OLK patients aged below 50 years old in the non-smoking group. The median age of the smoking group was significantly higher than that of the non-smoking group. Possibly, chronic oral inflammation occurs due to long-term smoking and chronic stimulation of the toxic substances in cigarettes, leading to defensive hyperplasia^18^. As a result, minor mucosal changes are perceptually invisible, and the patients are often older at the time of treatment. For non-smokers, the oral mucosa is sensitive, and mild adverse stimulation is more likely to induce OLK, so they visit hospitals early. There were more women among non-smokers. Probably, women are susceptible to OLK because of female hormone levels and physiological structure. OLK frequently occurs in the tongue and mouth floor^19^. In this study, the tongue and mouth floor were more prone to lesions in patients with abnormal hyperplasia in smoking and non-smoking groups. Moreover, moderate-severe abnormal hyperplasia was dominant in the smoking group, which was much severer than that in the non-smoking group. Therefore, the risk of malignant lesions should be well considered in the clinical diagnosis of smokers.

The expression of mTOR has been closely related to the hyperplasia degree of OLK, but it has no definite relationship with smoking history^20^. In this study, however, the number of cigarettes per day was not significantly related to Beclin-1 expression (*r*=-0.811, P=0.063), but significantly positively associated with mTOR expression (*r*=0.843, P=0.042). No correlation was observed between years of smoking and expressions of Beclin-1 and mTOR in the abnormal hyperplasia group. After the simple hyperplasia group was included, there was a positive correlation between years of smoking and positive expression rate of mTOR (*r*=0.942, P=0.012). Hence, with increasing amount of smoking, harmful substances in cigarettes further stimulated the oral cavity. Such stimulation promotes the PI3K/AKT/mTOR signalling pathway, thereby reducing the autophagy activity of cells. Besides, based on the number of cigarettes and years of smoking, the positive expression rates of Beclin-1 and mTOR had significant negative correlations with each other (*r*=-0.952, P=0.003; *r*=-0.953, P=0.002), showing significant differences (P<0.05). Taken together, with increasing number of cigarettes or duration of action, the decreased autophagy activity raises the degree of malignancy of tissues, thus elevating the risk. Therefore, OLK can be prevented and treated by stopping smoking.

In conclusion, autophagy-related proteins Beclin-1 and mTOR may be involved in the smoking-induced pathological process of OLK. Nevertheless, this study still has limitations. First, it was a retrospective study, and the clinical parameters were distributed unevenly, with a certain bias. Second, the sample size was small, which might lead to deviations in the results. In the future, the sample size will be expanded, and prospective studies will be conducted to clarify the impact of smoking on OLK, paving the way for the clinical diagnosis and treatment of OLK.
